# Towards a Treatment for Gulf War Illness: A Consensus Docking Approach

**DOI:** 10.1093/milmed/usz299

**Published:** 2020-02-19

**Authors:** Rajeev Jaundoo, Jonathan Bohmann, Gloria E Gutierrez, Nancy Klimas, Gordon Broderick, Travis J A Craddock

**Affiliations:** 1 Institute for Neuro-Immune Medicine, Nova Southeastern University, 3301 College Avenue, Fort Lauderdale, FL 33314-7796; 2 Department of Psychology & Neuroscience, Nova Southeastern University, 3301 College Avenue, Fort Lauderdale, FL 33314-7796; 3 Department of Clinical Immunology, Nova Southeastern University, 3301 College Avenue, Fort Lauderdale, FL 33314-7796; 4 Pharmaceuticals and Bioengineering Department, Southwest Research Institute, 6220 Culebra Road, San Antonio, TX 78238-5166; 5 Pharmaceuticals and Bioengineering, Chemistry and Chemical Engineering Division, Southwest Research Institute, 6220 Culebra Road, San Antonio, TX 78238-5166; 6 Miami Veterans Affairs Medical Center, 1201 N.W. 16th Street, Miami, FL 33125; 7 Rochester Institute of Technology, One Lomb Memorial Drive, Rochester, NY 14623-5603; 8 Centre for Clinical Systems Biology, Rochester General Hospital Research Institute, 100 Kings Highway South, Rochester, NY 14617; 9 Department of Computer Science, Nova Southeastern University, 3301 College Avenue, Fort Lauderdale, FL 33314-7796

## Abstract

**Introduction:**

Gulf War Illness (GWI) currently has no known cure and affects soldiers deployed during the Persian Gulf War. It is thought to originate from exposure to neurotoxicants combined with battlefield stress, and previous research indicates that treatment first involves inhibition of interleukin-2 and tumor necrosis factor alpha, followed by the glucocorticoid receptor. However, the off-target effects of pharmaceuticals hinder development of a drug treatment therapy.

**Materials and Methods:**

AutoDock 4.2, AutoDock Vina, and Schrodinger’s Glide were used to perform consensus docking, a computational technique where pharmaceuticals are screened against targets using multiple scoring algorithms to obtain consistent binding affinities. FDA approved pharmaceuticals were docked against the above-mentioned immune and stress targets to determine a drug therapy for GWI. Additionally, the androgen and estrogen targets were screened to avoid pharmaceuticals with off-target interactions.

**Results:**

While suramin bound to both immune targets with high affinity, top binders of the hormonal and glucocorticoid targets were non-specific towards their respective proteins, possibly due to high structure similarity between these proteins.

**Conclusions:**

Development of a drug treatment therapy for GWI is threatened by the tight interplay between the immune and hormonal systems, often leading to drug interactions. Increasing knowledge of these interactions can lead to break-through therapies.

## Introduction

Gulf War Illness (GWI) is a chronic multi-symptom illness with no known cure characterized by fatigue, musculoskeletal pain, gastrointestinal, and cognitive dysfunction believed to be a result of multiple chemical exposure to soldiers deployed to the theater of the 1990–1991 Persian Gulf War. ^1–4^ Pharmaceuticals tend to bind to multiple sites beyond their intended targets,^5^ leading to off-target interactions and/or adverse drug reactions, which pose a major concern for the already taxed systems of those with GWI. A major hypothesis of GWI pathophysiology proposes that toxicant exposure, aggravated by stress, triggers a neuroinflammatory cascade leading to altered homeostatic regulation.^1–3^ And consistent with symptoms of GWI such as musculo-skeletal pain and fatigue, this neuroinflammatory cascade extends outside the central nervous system to affect the immune and endocrine systems as well, which are both linked to the brain via the hypothalamic–pituitary–adrenal (HPA) axis. Golier et al.^6^ has reported HPA dysregulation in soldiers with GWI, supporting this hypothesis. To address this issue, Craddock et al.^7^ utilized discrete logic models to determine a treatment course that would correct the altered homeostatic regulation in individuals with GWI. This multi-intervention treatment course comprised of inhibiting Th1 immune cytokines interleukin-2 (IL-2) and tumor necrosis factor alpha (TNF-α), directly followed by inhibition of the glucocorticoid receptor (GCR), involved with the stress response; however, a specific pharmaceutical combination for this treatment course has yet to be determined. Here, the drug docking programs AutoDock 4.2 (AD4),^8^ AutoDock Vina 1.1.2 (VINA),^9^ and Schrodinger’s Glide 2016-4 (GLIDE)^10^ were used to identify FDA-approved drugs specific to each IL-2, TNF-α, and GCR. Due to the tight regulation between the hormonal and immune systems,^11^ the androgen (AR) and estrogen (ER) targets were also screened to ensure that only drugs specific to IL-2, TNF- α, and GCR were chosen, reducing the chances of off-target interactions. FDA-approved drugs were specifically used because their toxicity and efficacy have already been extensively profiled, they are readily available for in vitro testing, and the development of novel compounds is expensive in both time and cost.

## Methods

### Crystal Structure Preparation

Crystal structures of the AR (2 am9, 2amb, 2pnu), ER (4ivy, 4iw6, 4ivy), GCR (1nhz, 3 h52, 4mdd), IL-2 (1 m48, 1 m49), and TNF-α (4twt) targets were obtained from the RCSB Protein Data Bank (PDB).^12^ These crystal structures were chosen primarily on their amino acid sequence completeness and resolution (3 Å or less). Furthermore, only structures in complex with a small molecule binder, which can be either a drug (eg, mifepristone for GCR) or an endogenous ligand (eg, testosterone for AR or estrogen for ER), were chosen. This served two purposes; first, the small molecule’s crystallographic position on each target was used as the binding site, and second, it allowed for re-docking, a process in which the small molecule is docked back to its target. The small molecule’s docked pose should be within 2.0 Å of its original crystallographic one, verifying the docking program used can accurately reproduce the in vitro derived crystal structure. Following Garcia-Sosa and Maran’s^13^ study, crystal structures for all docking programs were prepared using the Protein Preparation Prepwizard^14^ tool (PrepWiz), which removed waters, added hydrogens, set charges, and adjusted bond orders. Epik,^15^ a pK(a) predictor, was utilized in tandem with PrepWiz to perform tautomerization. The *prepare_receptor4.py* utility from AutoDockTools 1.5.6^8^ added Gasteiger charges and converted the crystal structures to the PDBQT format required for AD4 and VINA.

### Ligand Preparation

1,794 FDA-approved drug structures were obtained from DrugBank’s February 15, 2016 database.^16–19^ The ligand preparation^20^ tool was used to prepare all drugs and add hydrogens for GLIDE. For AD4 and VINA, the ligands were converted to the PDB format using Open Babel 2.3.2;^21^ hydrogens, Gasteiger charges, and rotatable bonds were assigned using the AutoDockTools 1.5.6^8^ utility, *prepare_ligand4.py*.

### Crystal Structure Binders

To verify protocol accuracy, all small molecule binders were re-docked to their respective targets using each docking program. Next, the root mean squared deviation (RMSD) between the docked and crystallographic poses were calculated, which measures the how different the two poses are from one another. If AD4, VINA, or GLIDE failed to dock a known binder within a cutoff score of 2.0 Å, then that program was used for that crystal structure (Table I). To accurately compare RMSD between docked and crystallographic poses, AmberTools16^22^ was first used to normalize the atom numbers within the output files for all docked poses, which can change depending on docking program used.

**TABLE I:** Programs Matrix

	AD4	VINA	GLIDE
AR (2 am9)	*	*	*
AR (2amb)	*		
AR (2pnu)			*
ER (4ivy)			*
ER (4iw6)	*	*	*
ER (4iwf)			
GCR (1nhz)	*	*	*
GCR (3h52)	*	*	*
GCR (4mdd)	*	*	*
IL-2 (1m48)	*	*	*
IL-2 (1m49)	*	*	*
TNF-α (4twt)	*	*	

‘*’ signifies docking programs included in the final results. The RMSDs are: AR 2 am9: AD4 = 1.570, VINA = 1.334, GLIDE = 0.270; AR 2amb: AD4 = 1.948, VINA = 3.535, GLIDE = 3.979; AR 2pnu: AD4 = 7.654, VINA = 9.519, GLIDE = 1.576; ER 4ivy: AD4 = 4.052, VINA = 4.224, GLIDE = 1.242; ER 4iw6: AD4 = 0.921, VINA = 1.712, GLIDE = 0.519; ER 4iwf: AD4 = 4.146, VINA = 4.124, GLIDE = 4.056; GCR 1nhz: AD4 = 1.064, VINA = 0.970, GLIDE = 0.665; GCR 3 h52: AD4 = 1.113, VINA = 0.480, GLIDE = 0.831; GCR 4mdd: AD4 = 1.140, VINA = 0.779, GLIDE = 0.875; IL-2 1 m48: AD4 = 1.745, VINA = 2.019, GLIDE = 1.356; IL-2 1 m49: AD4 = 1.225, VINA = 1.650, GLIDE = 1.700; TNF-α 4twt: AD4 = 1.060, VINA = 1.174, GLIDE = NA (failed to dock).

### Docking & Post-Processing

Virtual screening (VS) was performed using the Pegasus supercomputer located at the University of Miami. Drug docking was completed using Python and Bash scripts that implemented Garcia-Sosa and Maran’s^13^ protocol for AD4, VINA, and GLIDE. That being said, this protocol was adjusted, so that the Coulomb and van der Waals interaction energy cutoff score (CV cutoff) was set to ‘9999.9’ to report all binding energies, even positive, and a single docking run was performed with Schrodinger’s more extensive standard-precision (SP) scoring function instead of high-throughput VS (HTVS) scoring function. These scoring functions are based on the amount of central processing unit time required; HTVS is designed for quick preliminary screenings, while SP is intended for large databases of compounds. Once VS was completed, only each ligand’s lowest energy pose from AD4, VINA, and GLIDE were used in further analysis.

To rank the results, the median absolute deviation from the median (MADM) of each ligand’s pose was calculated from AD4, VINA, and GLIDE from all crystal structures of a given target. The MADM formula is as follows:^23^

$\mathrm{MADM}=\mathrm{median}\Big(\Big|{X}_i-\mathrm{median}(X)\Big|\Big),\kern0.36em i=1..N$

Where *X_i_* refers to the *i*^th^ free binding energy of the pose to the crystal structure, and *X* refers to all *N* binding energies from all docking programs from all crystal structures. In contrast to the standard deviation and mean, the MADM is not skewed by outliers, and is able to discern outlier values even when the sample size is small.^23^ The MADM was used due to this robustness, especially when scoring a wide variety of binding energies. The upper and lower bounds were determined using the formula:

$\Big[{X}_{\mathrm{lower}},{X}_{\mathrm{upper}}\Big]=\mathrm{median}(X)\pm \Big(3.5\times \mathrm{MADM}\Big)$

Wanting to be as inclusive as possible, only outlier energies that were greater than a threshold of 3.5 absolute deviations around the median were eliminated. Once these values were removed, the free binding energy of all poses predicted from AD4, then VINA, and finally GLIDE were ranked from lowest/best to highest/worst. This initial ranking process was performed separately for each docking program. Next, to obtain the sum of ranks (SoR), we added the ranks from all three docking programs together. For example:

If AD4 rank = 10, VINA rank = 15, GLIDE rank = 12: SoR = 10 + 15 + 12 = 37.

Note that if a program failed to dock the ligand, then a value of NaN was used, which is equal to 0 when computing the SoR (ie AD4 = 1, VINA = NaN, and GLIDE = 3, then SoR = 4). Finally, the results were ordered lowest (best) to highest (worst) SoR, and that order is considered the overall rank (OR). Note that one limitation of the SoR is that a value of 0 from any number of docking programs would skew the results. That being said, the OR for known drugs such as testosterone for AR and mifepristone for GCR were within the top 10 binders of their respective targets. The high ranking and binding affinities of these known binders were reflective of in vitro results, endorsing the OR metric. The top 10 hits for each target are shown in Tables II to VI.

**FIGURE 1:**
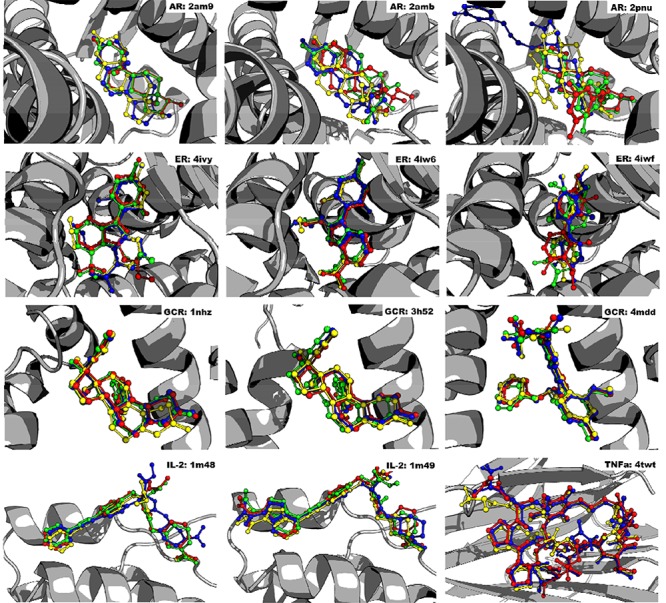
Redocking of known binders. Known binder compared to AD4, VINA, and GLIDE. Note that residues 636–652 of GCR were removed for clarity. All images were created using PyMOL version 1.8.6.2.^19^ The known binders for each target are listed as follows: AR 2am9: TES, AR 2amb: 17H, AR 2pnu: EMN, ER 4ivy:1GT, ER 4iw6: 1GU, ER 4iwf: 15Q, GCR 1nhz: 486, GCR 3h52: 486, GCR 4mdd: 29M, IL-2 1m48: FRG, IL-2 1m49: CMM, TNF-α 4twt: ALA-CYS-PRO-PRO-CYS-LEU-TRP-GLN-VAL-LEU-CYSGLY.

**TABLE II:** Top 10 IL-2 Results

OR	Ligand	SoR	Mean ± SD	NNT	NNT: mean ± SD	*P*-value
1	Oritavancin	24	−12.09 ± 2.95	TNF-α: 45	−10.63 ± 2.83	0.70
2	Vapreotide	65	−11.54 ± 3.43	TNF-α: 7	−11.17 ± 2.07	0.91
3	Suramin	120	−9.52 ± 1.36	TNF-α: 2	−12.39 ± 2.49	0.45
4	Amphotericin-B	121	−8.72 ± 2.13	TNF-α: 17	−10.20 ± 0.40	0.20
5	Nystatin	142	−9.05 ± 1.53	TNF-α: 39	−9.39 ± 0.09	0.64
6	Bacitracin	153	−9.46 ± 1.43	GCR: 414	−7.76 ± 1.70	0.17
7	Felypressin	172	−8.65 ± 1.44	TNF-α: 167	−7.99 ± 0.19	0.36
8	Sirolimus	199	−7.16 ± 3.10	TNF-α: 22	−10.12 ± 0.12	0.09
9	Micafungin	233	−9.79 ± 3.82	TNF-α: 316	−11.54 ± 5.04	0.79
10	Rifapentine	238	−7.07 ± 2.74	TNF-α: 9	−10.56 ± 0.66	0.05

OR refers to the overall rank, SoR refers to the sum of ranks, and SD refers to the standard deviation.

Additionally, each ligand’s specificity to their intended target was determined using another metric known as the next nearest target (NNT). The NNT was determined by comparing a ligand’s rank on a given target to its rank on every other target. The target with the best/lowest rank for the current ligand is considered the NNT; or in other words, NNT is the target that the ligand would most likely bind to after the current one. For instance, to find the NNT of mifepristone on GCR, we examined its position on the other targets: AR = 1392, ER = 1525, IL-2 = 299, and TNF-α = 499. In this case, IL-2 was the NNT because it contained the best/lowest rank for mifepristone after GCR. More selective drugs had a greater difference between their rank on a given target and NNT. Additionally, a two-tailed *t*-test was performed to examine how well a ligand bound to a target by comparing its binding energy predictions across all docking programs to that of the NNT’s, in which case a significant value was considered to be ≤0.05. Statistically significant values meant that the ligand was more likely to bind to either the NNT or its intended target; one or the other. Non-significant *P*-values meant that the ligand was just as likely to bind to the NNT as the intended target, signifying the ligand was non-specific.

## Results

A total of 1,794 ligands were docked using AD4, VINA, and GLIDE to the targets AR, ER, GCR, IL-2, and TNF-α. For each crystal structure, only results from programs which docked known binders to within 2.0 Å of their crystallographic pose were computed, a cutoff known to reliably identify correctly docked ligands.^24^Table I displays which docking programs were omitted for which crystal structures. AutoDock 4.2 and VINA were excluded from AR 2pnu, ER 4ivy, and ER 4iwf because their predicted poses for the known binders were above the 2.0 Å RMSD cutoff range. Similar to AD4 and VINA, GLIDE was excluded from AR 2amb and ER 4iwf due to exceeding the RMSD cutoff score. GLIDE failed to predict a pose for TNF-α 4twt’s known binder altogether. A graphical representation of Table I’s information is shown in Figure 1. The *P*-values between the top 10 compounds for each target and their NNT were calculated, and the majority of top compounds were not specific to their targets, as evidenced by their *P*-values ≥0.05. Supplementary data contains a database with the full results.

For IL-2’s top ligands, sirolimus and rifapentine were marginally significant with values <0.10. The overwhelming majority of compounds for IL-2 had NNTs of TNF-α, and this trend continued for the top 20 compounds as well, with the exception of tavaborole (rank 11), whose NNT was GCR.

TNF-α’s top ligands included two *P*-values of ‘NA’: sucralfate had no NNT since it failed to bind to any other target, and cyclosporine’s standard deviation of 0 made a *t*-test impossible. This left rifapentine as the marginally specific ligand for TNF-α, similar to IL-2. Dactinomycin only bound to TNF-α; it failed to bind to AR, ER, GCR, and IL-2 as evidenced by the abnormally high mean and standard deviation of its NNT. This may have been due to the large size of dactinomycin (C_62_H_86_N_12_O_16_), which prevented it from docking to smaller binding regions. The most popular NNT structure for TNF-α’s top 10 and top 20 ligands was IL-2. Outliers included ledipasvir (rank 13, NNT: ER), Ergotamine (rank 15, NNT: GCR), and amphotericin-B (rank 17, NNT: AR).

Within GCR’s top ligands, antrafenine, mifepristone, pimozide, and vilazodone were shown to be the most specific binders. The immune targets TNF-α and IL-2 were the most prevalent NNTs for the majority of GCR’s top 10 and top 20 ligands.

**TABLE III:** Top 10 TNF-α Results

OR	Ligand	SoR	Mean ± SD	NNT	NNT: mean ± SD	*P*-value
1	Sucralfate	1	−20.16 ± 0.00	NA	NA	NA
2	Suramin	19	−12.39 ± 2.49	IL-2: 3	−9.52 ± 1.36	0.45
3	Vancomycin	23	−12.20 ± 2.90	IL-2: 52	−8.93 ± 3.94	0.45
4	Lanreotide	35	−11.34 ± 2.04	ER: 460	−7.11 ± 1.92	0.23
5	Cyclosporine	35	−8.80 ± 0.00	GCR: 257	−3.79 ± 0.61	NA
6	Porfimer	42	−12.68 ± 4.08	IL-2: 150	−8.94 ± 2.89	0.52
7	Vapreotide	43	−11.17 ± 2.07	IL-2: 2	−11.54 ± 3.43	0.91
8	Dactinomycin	53	−11.42 ± 2.72	GCR: 1021	1778.87 ± 2117.24	0.12
9	Rifapentine	56	−10.56 ± 0.66	IL-2: 10	−7.07 ± 2.74	0.05
10	Desmopressin	62	−10.32 ± 1.12	ER: 15	−8.61 ± 0.71	0.34

OR refers to the overall rank, SoR refers to the sum of ranks, and SD refers to the standard deviation.

**TABLE IV:** Top 10 GCR Results

OR	Ligand	SoR	Mean ± SD	NNT	NNT: mean ± SD	*P*-value
1	Curcumin	209	−8.10 ± 0.51	ER: 139	−7.08 ± 1.85	0.42
2	Antrafenine	220	−9.82 ± 1.12	IL-2: 49	−6.74 ± 2.10	0.02
3	Mifepristone	245	−9.49 ± 2.30	IL-2: 299	−5.55 ± 1.36	0.02
4	Indinavir	256	−10.38 ± 2.69	TNF-α: 58	−9.40 ± 1.60	0.65
5	Nebivolol	523	−9.83 ± 1.58	AR: 38	−9.55 ± 1.97	0.81
6	Darifenacin	586	−9.44 ± 1.49	TNF-α: 66	−8.82 ± 0.08	0.28
7	Norethindrone	646	−7.68 ± 0.17	ER: 427	−7.60 ± 1.03	0.89
8	Pimozide	656	−9.74 ± 0.92	TNF-α: 89	−8.51 ± 0.09	0.02
9	Bazedoxifene	686	−9.80 ± 1.41	TNF-α: 126	−8.47 ± 0.97	0.37
10	Vilazodone	713	−8.97 ± 1.13	IL-2: 38	−7.09 ± 1.44	0.04

OR refers to the overall rank, SoR refers to the sum of ranks, and SD refers to the standard deviation.

**TABLE V:** Top 10 AR Results

OR	Ligand	SoR	Mean ± SD	NNT	NNT: mean ± SD	*P*-value
1	Digoxin	24	−9.03 ± 1.65	TNF-α: 21	−10.54 ± 2.24	0.63
2	Brexpiprazole	26	−7.70 ± 3.80	ER: 14	−7.86 ± 2.46	0.95
3	Docetaxel	67	−8.25 ± 1.60	ER: 117	−7.58 ± 0.68	0.64
4	Dexibuprofen	73	−8.31 ± 0.92	IL-2: 374	−6.10 ± 0.98	0.01
5	Testosterone	81	−8.67 ± 1.93	ER: 22	−9.06 ± 0.70	0.73
6	Haloperidol	91	−8.37 ± 1.53	IL-2: 115	−6.27 ± 1.51	0.07
7	Estrone	110	−9.47 ± 0.31	ER: 19	−9.17 ± 0.70	0.52
8	Fenoprofen	116	−8.77 ± 0.38	IL-2: 424	−6.08 ± 1.11	0.00
9	Estradiol	174	−8.52 ± 1.19	ER: 16	−9.15 ± 0.55	0.39
10	Benzylpenicillin	207	−3.68 ± 6.61	IL-2: 726	−5.46 ± 0.92	0.62

OR refers to the overall rank, SoR refers to the sum of ranks, and SD refers to the standard deviation.

**TABLE VI:** Top 10 ER Results

OR	Ligand	SoR	Mean ± SD	NNT	NNT: mean ± SD	*P*-value
1	Demeclocycline	69	−9.59 ± 0.29	AR: 30	−6.87 ± 0.76	0.02
2	Paroxetine	73	−9.71 ± 0.38	AR: 54	−7.59 ± 0.70	0.00
3	Butoconazole	142	−8.68 ± 1.45	GCR: 130	−8.60 ± 0.67	0.93
4	Oxazepam	194	−8.62 ± 0.38	GCR: 471	−7.85 ± 0.62	0.36
5	Setiptiline	206	−9.41 ± 0.18	IL-2: 364	−5.65 ± 1.09	0.00
6	Pergolide	206	−8.56 ± 1.15	AR: 173	−7.72 ± 1.70	0.46
7	Mequitazine	210	−8.46 ± 1.40	TNF-α: 594	−7.03 ± 0.23	0.18
8	Equilin	217	−9.28 ± 0.46	AR: 23	−8.60 ± 0.89	0.23
9	Trazodone	227	−8.64 ± 1.06	GCR: 173	−8.49 ± 0.61	0.84
10	Ospemifene	239	−9.40 ± 0.76	GCR: 386	−8.05 ± 0.74	0.04
16	Estradiol	292	−9.15 ± 0.55	AR: 9	−8.52 ± 1.19	0.39

OR refers to the overall rank, SoR refers to the sum of ranks, and SD refers to the standard deviation.

In regards to AR’s top ligands, dexibuprofen and fenoprofen were the two significant binders with haloperidol as marginally significant. ER was the dominant NNT structure for the top 10 and top 20 ligands, with IL-2 as the next most common NNT.

Finally, ER’s top ligands contained a considerable number significant binders such as demeclocycline, paroxetine, setiptiline, and ospemifene. Note that estradiol was included in Table VI due to its role as one of ER’s known binders. AR was the most common NNT, followed by GCR, for the top 10 and 20 ligands. However, within ER’s top 20 ligands, there were two exceptions to the rule. Mirabegron’s (rank 13) NNT was IL-2 and desmopressin’s (rank 15) NNT was TNF-α.

## Discussion and Conclusions

Previous work^7^ has implicated immune and hormone dysregulation in GWI, and through drug repurposing, these same interactions may be leveraged towards drug treatment therapies. Here, a non-filtering strategy of consensus docking was utilized to ameliorate the problem of over filtration, where significant off-target interactions are too often neglected, leading to side effects during treatment. Consensus docking of FDA approved drugs using AD4, VINA, and GLIDE was performed on immune (TNF-α, IL-2) and stress-related (GCR) targets to find pharmaceuticals that specifically bound these targets, which would correct the altered homeostatic regulation in individuals with GWI. And due to the tight interplay between the immune and hormonal systems, the hormonal targets (AR, ER) were additionally screened to avoid pharmaceuticals that bound to both hormonal and immune targets; in other words, pharmaceuticals with off-target interactions were avoided. Ensuring our protocol reflected results from in vitro experiments, only results from docking programs that re-docked their small molecule binder within 2.0 Å of its original pose were calculated. Furthermore, the NNT metric was developed to determine how specific each pharmaceutical was to their intended target, where larger discrepancies between a pharmaceuticals’ rank on its intended target and its NNT signaled greater specificity. The MADM bound equation was applied to all pharmaceuticals for every target, and any value beyond 3.5 standard deviations above/below the median binding energy was removed. The results were then ordered based on the SoR for all docking programs.

As this study is purely computational in nature, it does require further experimental validation. However, comparison against current literature does highlight some potential leads. Literature supports that the top 10 compounds identified to bind with IL-2 have the potential to directly bind to this target. Previous studies have shown that in vivo, suramin inhibits IL-2 binding to the IL-2 target in a concentration dependent manner.^25^ And although no literature currently exists demonstrating the binding affinity of antifungal agents to IL-2 directly, amphotericin-B, nystatin, and micafungin have shown to express immunomodulatory properties by stimulating the production of cytokines such as IL-1, IL-8, IL-10, and TNF-α.^26^^,^^27^ While sirolimus reduces T-cell and B-cell sensitivity to IL-2, the primary mode is through mTOR inhibition,^28^ and there were no known studies found examining whether sirolimus directly binds to IL-2. Likewise, there is currently no literature which investigates the in vitro binding of oritavancin, bacitracin, felypressin, and rifapentine to IL-2. That being said, rifamycins (eg. rifapentine, rifampicin, rifadin, etc.) have been shown to be immunosuppressive, although the mechanisms behind this behavior are unknown.^29^

Similar to IL-2, current literature only shows one of TNF-α’s top hits as an experimental verified binder. Suramin inhibits the bioactivity of TNF-α by directly binding to TNF-α.^30^ Overall, all of TNF-α’s top compounds were found to be inhibitory. For instance, the antibiotics vancomycin and rifapentine also double as immunomodulators, which affect TNF-α pathways as well as signaling.^29^^,^^31^ Additionally, studies have shown that sucralfate, an anti-inflammatory agent, regulates the expression of TNF-α in rats, although the mechanism is not known at this time.^32^ Interestingly, Wafa et al.^33^ has shown that desmopressin decreased TNF-α plasma levels during experimental treatment of endotoxemia, implicating its anti-inflammatory properties. Finally, no studies were found that documents the direct binding effects of cyclosporine, lanreotide, vapreotide, dactinomycin, or porfimer to TNF-α, although immune modulation by these drugs has been found. Note that dactinomycin only bound to the TNF-α protein, which explains the low ranking of its NNT.

AR, ER, GCR, and the progesterone receptor (PR) are part of the nuclear receptor family, which among other things, have similar protein structures.^34^ While curcumin modulates GCR transcription,^35^ curcumin analogues can operate as 17α-substituted dihydrotestosterone, an AR antagonist.^36^ Likewise, mifepristone is both an PR and GCR antagonist,^37^ nebivolol functions as an antagonist for nuclear receptors AR, ER, and PR,^38^ while norethindrone behaves as a synthetic progesterone.^16^^,^^17^ Bazedoxifene binds to ER,^39^ whose protein structure is similar to that of GCR and AR. No definitive information was available for antrafenine, indinavir, darifenacin, pimozide, or vilazodone regarding their ability to agonize/antagonize GCR.

Four out of the top 10 ligands for AR are experimentally shown to be direct binders. The top hit, digoxin, acts as an estrogen-like molecule under certain conditions,^40^ and can prohibit association of testosterone conjugates to membrane ARs.^41^ In addition to both being hormonal targets, the crystal structures used in this study for AR and ER are very similar, which may account for the binding of estrogens. That being said, experimental data have shown that estrone and estradiol are in fact binders of AR, albeit with lower affinity than androgens such as testosterone.^42^ Furthermore, fenoprofen and docetaxel both downregulate AR expression and signaling,^43^^,^^44^ and although no studies have shown that fenoprofen and dexibuprofen directly bind to AR, both of their pharmacological profiles are very similar to aspirin,^16^^,^^17^^,^^45^ which can inhibit androgen’s response to human chorionic gonadotropin.^46^ Lastly, no current literature supports brexpiprazole, haloperidol, or benzylpenicillin as AR agonists/antagonists.

For ER, four of the top 10 compounds were experimentally validated as direct binders. Paroxetine is an antidepressant that functions as an ER agonist,^47^ imidazoles (eg. butoconazole) have bound to ER in vitro,^48^ and selective ER modulators such as ospemifene that can mimic the effects of estrogens.^49^^,^^50^ Equilin was another top binder due to its origins as an estrogen related steroid, and the antidepressant setiptiline has been found to interact with ER in varying degrees.^51^ No literature was found that suggests pergolide, demeclocycline, oxazepam, mequitazine, or trazodone affects ER directly.

In conclusion, GWI is thought to originate from exposure to battlefield neurotoxicants and then further exacerbated by stress, requiring a treatment strategy that inhibits both immune and stress-related targets. However, pharmaceuticals on average bind to at least six different targets,^5^ which is why the focus on off-target interactions is a must to avoid adverse drug effects during treatment. The consensus docking method used here takes into account off-target effects of pharmaceuticals by utilizing the NNT metric, which measures a drug’s specificity to their intended target. This not only leads to the development of treatments with fewer side effects, but also understanding how each pharmaceutical interacts within the body may lead to a more effective drug therapy overall.

## Notes

Presented as a poster at the 2018 Military Health System Research Symposium, August 2018, Kissimmee, FL; abstract no. MHSRS-18-1919.

## Notes

The opinions and assertions contained herein are the private views of the authors and are not to be construed as official or as reflecting the views of the Department of Defense. The authors indicate that they do not have any conflicts of interest.
